# Methyl vinyl ketone impairs spatial memory and activates hippocampal glial cells in mice

**DOI:** 10.1371/journal.pone.0289714

**Published:** 2023-08-31

**Authors:** Ren Y. Sato, Koki Kotake, Yumin Zhang, Hiraku Onishi, Futaba Matsui, Hiroaki Norimoto, Zhiwen Zhou

**Affiliations:** Graduate School of Medicine, Hokkaido University, Sapporo, Japan; Nathan S Kline Institute, UNITED STATES

## Abstract

Memory is a fundamental brain function that can be affected by a variety of external factors including environmental pollutants. One of these pollutants is methyl vinyl ketone (MVK), a hazardous substance found in cigarettes, industrial wastes, and car exhaust. Humans can be exposed to MVK under many circumstances; however, it is unclear whether MVK affects higher-order brain functions such as memory. Here, we examined the memory performances of mice receiving systemic MVK administration. We found that 1 mg/kg of MVK impaired spatial memory. We also showed that 1 mg/kg MVK activated glial cells and altered glial functions in several subregions of the hippocampus, a brain region involved in learning and memory. These results suggest that MVK induces memory deficits and activates glial cells in hippocampal subregions.

## Introduction

Memory is a fundamental brain function that originates from the refined and intricate communications among a vast number of neurons and glial cells. It is susceptible to chemical stimuli caused by food, drugs, and environmental substances. Multiple environmental pollutants including tetraethyllead, PM2.5, and acrolein have been demonstrated to have a detrimental impact on memory performance [1–3].

**Fig 1:**
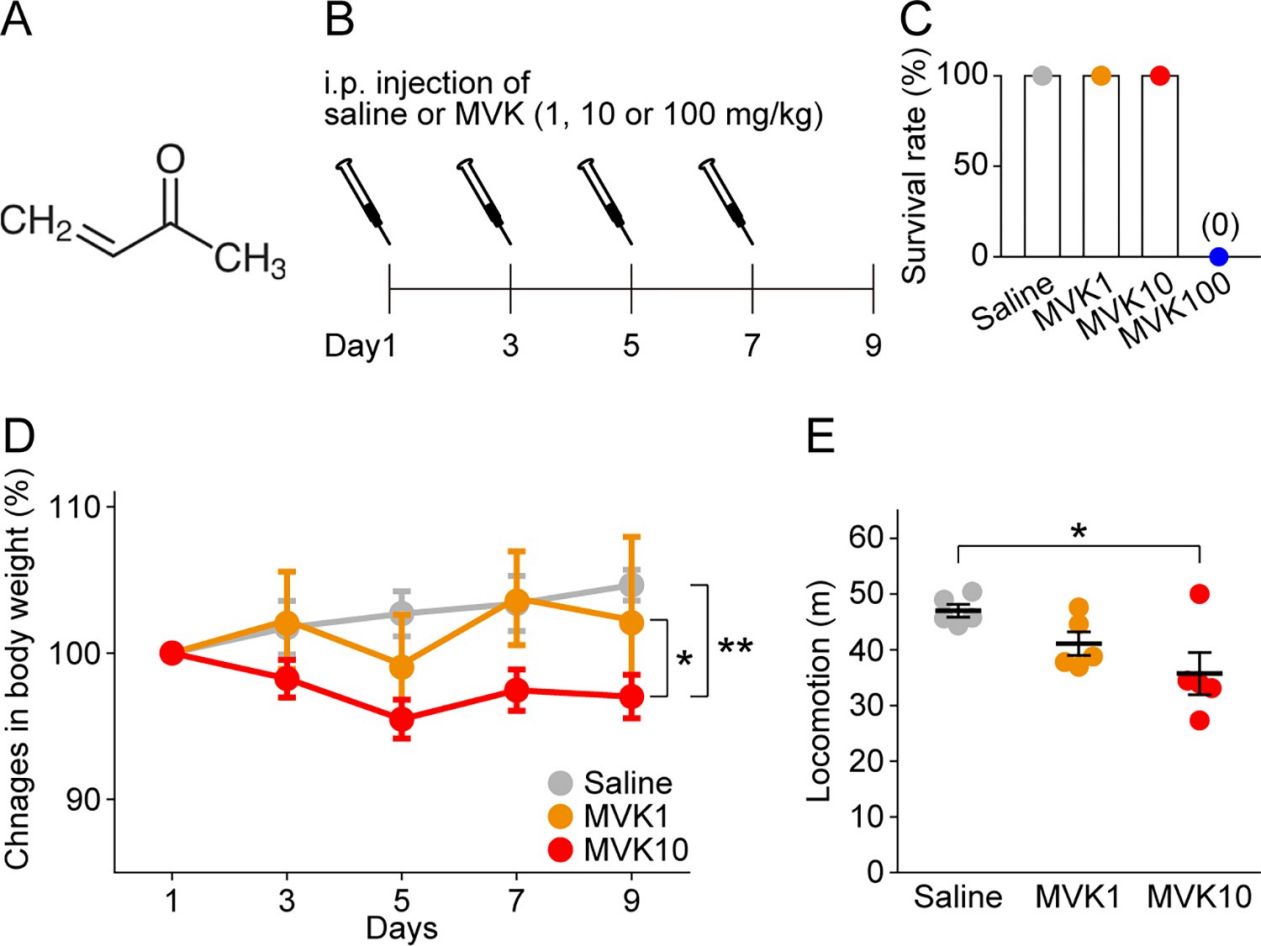
The influence of different dosages of methyl vinyl ketone (MVK) administration. (A) The chemical structure of MVK. (B) The time course of saline or MVK administration. Bodyweights of mice were measured on the day of administration and 2 days after the last administration. (C) The survival rate of mice administered saline or MVK. N = 5 for Saline, MVK1, and MVK10 mice. N = 3 for MVK100 mice. (D) Percentage changes in bodyweight gain of Saline, MVK1, and MVK10 mice. N = 5 for each group. (E) The distance mice traveled exploring the transparent box in 5 min. N = 5 for each group. *p < 0.05, **p < 0.01. The data are presented as mean ± standard error of the mean (SEM). Small dots indicate individual data points of each mouse. MVK1 = 1 mg/kg MVK, MVK10 = 10 mg/kg MVK, MVK100 = 100 mg/kg MVK, i.p. = intraperitoneal.

One of the environmental pollutants is methyl vinyl ketone (MVK). MVK is an oxidant and alkylating agent from α, β-unsaturated carbonyl group [4] (Fig 1A). Humans can be exposed to MVK in many ways. Smoking may be the most common example of MVK uptake since higher levels of MVK metabolites are found in the urine of smokers compared to nonsmokers [5]. MVK is also released into the environment as automobile exhaust and industrial wastes due to its use in commercial products [6]. Therefore, it is unlikely to avoid exposure to MVK completely.

Previous studies have examined the toxicity of MVK in several types of cells and tissues. Acute exposure to MVK is known to irritate eyes and skin [7], and chronic MVK inhalation has been shown to cause respiratory organ damage with immune activation in rats [8]. *In vitro* experiments have shown that MVK induced cell death of melanoma cells and GT1-7 cells in mice [9, 10]. Recently, our group has found that MVK increases the death rate of cultured hippocampal neurons and inhibits their axonal branching and outgrowth [11]. However, it is still unclear whether MVK affects brain functions or not.

In this study, we focused on the effects of MVK on spatial memory in mice. We found that intraperitoneal (i.p.) administration of MVK impaired spatial memory. We also found that MVK induced changes in astrocytes and microglia in several subregions of the hippocampus, a critical brain region involved in learning and memory [12]. Overall, our finding reveals that MVK causes spatial memory deficits and alters glial functions in the hippocampus.

## Materials and methods

### Animals and drug administration

We performed the experiments under the approval of the Hokkaido University Animal Experiment Committee (21–0092) according to the Hokkaido University’s guidelines for the care and use of laboratory animals. 7-week-old male C57BL6/J (SLC, Inc., Shizuoka, Japan) mice were used. During the experimental period, mice had free access to food and water.

We administered saline or MVK (i.p., 1 mg/kg, 10 mg/kg or 100 mg/kg; Tokyo Chemical Industry Co., Ltd., Tokyo, Japan) according to the time course shown in Fig 1B. Mice were weighed at the time of drug administration and 2 days after the last dose.

### Behavioral tests

Object-location recognition test was carried out according to previous research [13]. A square, transparent box (30 cm in width, 30 cm in length, and 30 cm in depth, with an open top) was used as the chamber for object-location recognition test. The chamber was placed in a stable environment with minimal changes during behavioral sessions. Mice were able to see the surroundings through the transparent walls. Mice were habituated to the chamber for 15 min per day for 2 days. During the training, mice explored the chamber for 10 min with two identical bowl-like objects placed around the corners.

During the test, mice explored the chamber for 5 min with one of the objects relocated. A camera was installed above the center of the chamber to record the sessions. Exploration time was defined as the time that the mouse spent sniffing or touching the objects. However, the time when the mouse was chewing or climbing the objects or accidentally touching them during grooming was excluded from the object exploration time. Memory was evaluated by the following discrimination score:

$$Discrimination\;score=\frac{\left(t_{a}-t_{b}\right)}{\left(t_{a}+t_{b}\right)}$$

*t*_*a*_ is the time mice spent exploring the relocated object, and *t*_*b*_ indicates the time mice spent exploring the other object.

To assess the locomotion of the mice, the distance they traveled exploring a transparent box in 5 min was calculated. First, the coordinates of the nose of mice were tracked using DeepLabCut (Mathis Laboratory, Lausanne, Switzerland). We then calculated the distance mice traveled using these coordinates.

### Immunohistochemistry

Mice were anesthetized with isoflurane inhalation and perfused transcardially with ice-cold PBS followed by 4% paraformaldehyde in 0.1 M PBS. After perfusion, brains were quickly removed and placed in 4% paraformaldehyde for one night. Subsequently, 100-μm thick coronal brain sections were made using a vibratome (VT1200, Leica Biosystems, Wetzlar, Germany).

Brain sections were permeabilized and blocked for nonspecific staining with 10% goat serum (Funakoshi, Tokyo, Japan) and 0.3% Triton X-100 (Sigma Aldrich, MO, USA) in PBS. For immunohistochemical detection of Iba1, CD68, GFAP, and NeuN, we used primary antibodies including rabbit anti-Iba1 antibody (1:500; Wako, Osaka, Japan), rat anti-mouse CD68 antibody (1:500; Bio-Rad, CA, USA), rabbit anti-GFAP antibody (1:1,000; Sigma Aldrich, MO, USA), and guinea pig anti-NeuN antibody (1:2,000; Synaptic Systems, Göttingen, Germany). Brain sections were incubated overnight at 4°C with primary antibodies. After washing three times with PBS, brain sections were incubated with the corresponding secondary antibodies (1:500; Goat anti-rat Alexa Fluor 488, Goat anti-rabbit Alexa Fluor 594, Goat anti-guinea pig Alexa Fluor 647, Thermo Fisher Scientific, MA, USA). After incubation overnight at 4° C, brain sections were washed three times with PBS. For nuclear staining with Hoechst (1:1,000; Invitrogen, MA, USA), brain sections were incubated in PBS with Hoechst for 15 min during the second wash after the secondary antibody staining.

### Acquisition and analysis of the immunostaining images

Images of the immunostained brain sections were acquired using an FV1000 confocal scanning microscope with a 20x objective lens (Olympus, Tokyo, Japan).

The neuron densities of the pyramidal cell layer of hippocampal CA1 and CA3 and the neuronal density of the dentate gyrus (DG) granule cell layer were calculated as follows. First, we counted the number of NeuN overlapping with Hoechst. Then, we measured the area of the pyramidal cell layers and the granule cell layer of the hippocampus.

The area of GFAP was calculated in the following manner. First, the threshold was set. Then, the percentage of GFAP positivity area was calculated in each hippocampal region.

The average fluorescence intensity of Iba1 within each hippocampal region was calculated. The density of microglia was calculated as follows: first, we set the thresholds for Iba1 and Hoechst; then, we counted the number of puncta larger than 19.28 μm that were labeled by both Hoechst and Iba1 fluorescence. For measuring the microglia and CD68 volume, first, the thresholds were set for Iba1 and CD68. Next, the Iba1 or CD68 positive area was calculated in 10 consecutive plains at 1 μm interval. The volume in each plain was summed. All thresholds were determined by minimizing the signals from non-specific binding and the loss of target signals. We conducted all analyses of immunostained images using Fiji software (NIH, MD, USA).

### Statistical analysis

We performed all statistical analyses using SigmaPlot 12.0 software (Systat Software Inc., CA, USA). When determining the statistical significance between two groups or against a fixed value, we first checked the normality of these data using the Shapiro-Wilk test and the equal variance test. If both tests are passed, we used Student’s *t*-test; otherwise, the Mann-Whitney Rank Sum Test was used. ANOVA was used to determine statistical significance among three or more groups. In the experiment examining bodyweight change, two-way ANOVA was used. In the memory test, one-way ANOVA was used. Tukey’s test was used in the post hoc analysis of ANOVA to determine statistical significance between each pair of groups.

## Result

### High-dose administration of MVK reduces bodyweight

We first examined the effects of MVK on the health of mice. Saline, 1 mg/kg MVK, 10 mg/kg MVK or 100 mg/kg MVK was administered intraperitoneally to mice once every two days for a total of four times (hereafter the mice received each administration are referred to as Saline mice, MVK1 mice, MVK10 mice or MVK100 mice respectively; Fig 1B). Mice were weighed before each drug administration and 2 days after the last administration (Fig 1B). All MVK100 mice died within 1 hour of the first MVK administration (Fig 1C). All other mice survived until the end of the experimental period. We calculated the change of bodyweight relative to the bodyweight before the first drug administration. Saline mice and MVK1 mice gradually gained weight during the experimental period. There was no significant difference in bodyweight change rate between Saline mice and MVK1 mice (*q* = 0.95, *p* = 0.78 by Tukey’s test after two-way ANOVA; Fig 1D). On the other hand, the bodyweight change rate of MVK10 mice decreased compared to Saline mice and MVK1 mice (Saline vs. MVK10, *q* = 4.6, *p* = 5.0×10^−3^; MVK1 vs. MVK 10, *q* = 3.7, *p* = 0.032 by Tukey’s test after two-way ANOVA; Fig 1D). We also assessed the locomotion of the mice during 5 min of exploration. MVK10 mice traveled a significantly shorter distance than Saline mice (Saline vs. MVK10, *q* = 4.4, *p* = 0.024 by Tukey’s test after one-way ANOVA; Fig 1E). These results suggest that the repeated i.p. administration of 10 mg/kg MVK undermined the health of mice.

### MVK inhibits spatial memory

**Fig 2:**
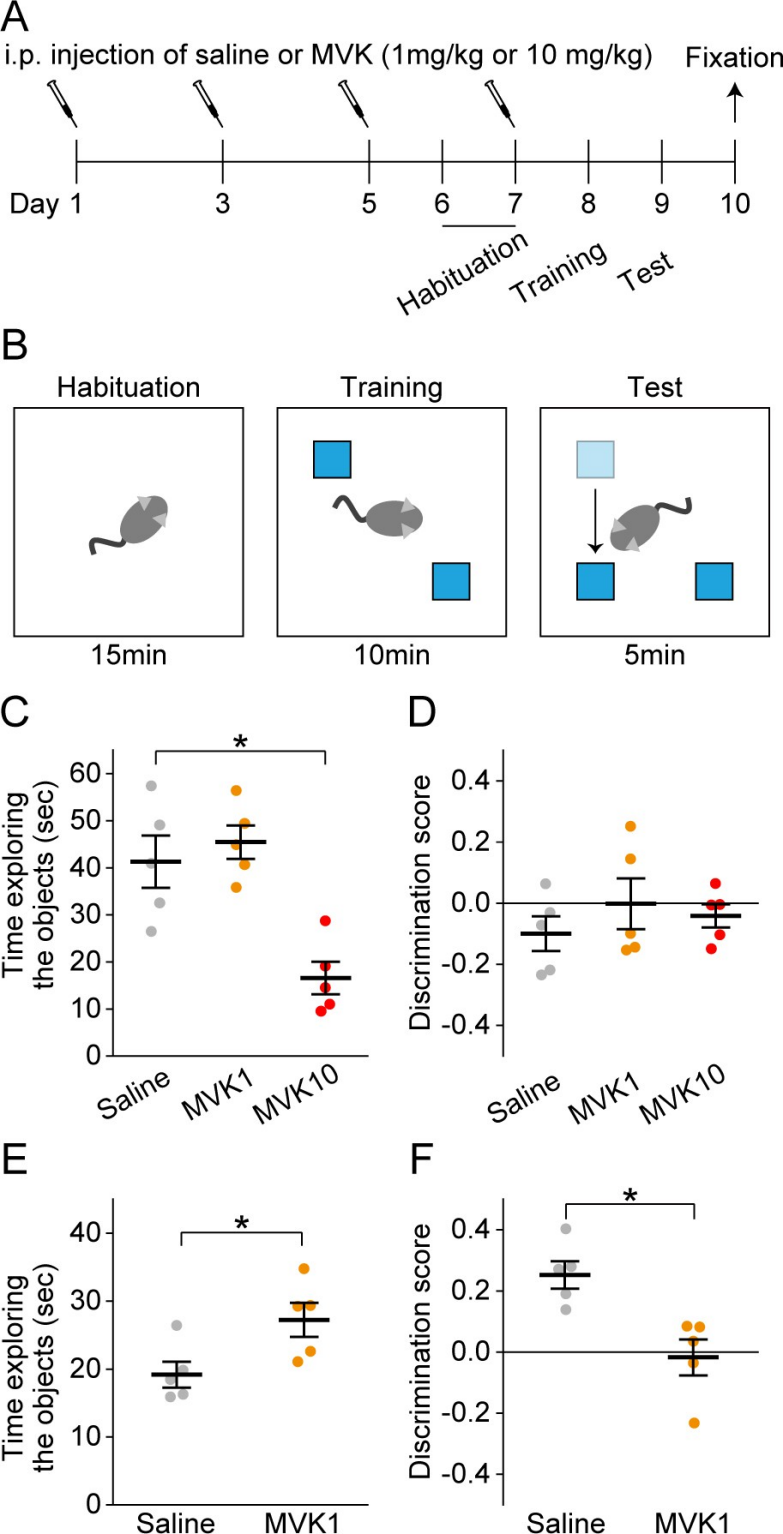
MVK impairs spatial memory. (A) The time course of the object-location recognition test. (B) Schematic diagram of the object-location recognition test. Blue boxes represent objects. (C) The time mice exploring the objects during the training session. N = 5 for each group. (D) The discrimination score during the training session. N = 5 for each group. (E) The time mice exploring the objects during the test session. N = 5 for each group. (F) The discrimination score during the test session. N = 5 for each group. *p < 0.05. The data are presented as mean ± SEM. Small dots indicate individual data points of each mouse.

Next, we examined the effects of MVK exposure on spatial memory (Fig 2A). In the object-location recognition test, the mice were first subjected to two identical objects in a familiar chamber during training sessions. Then, one of the objects was relocated and the mice explored the two objects again during the test sessions. Since mice are more likely to explore the relocated object if they can remember the locations of the objects, we can assess their memory of previous training by measuring the time they spent exploring the objects during test sessions [13]. The day after the second habituation, mice were subjected to training sessions followed by test sessions 24 hours later (Fig 2B). The test results were evaluated using the discrimination score.

During training sessions, discrimination scores were at chance level in all groups (Saline, *t* (4) = -1.8, *p* = 0.16; MVK1, *t* (4) = -9.3×10^−3^, *p* = 0.99; MVK10, *t* (4) = -1.1, *p* = 0.35 by one sample *t*-test; Fig 2D). MVK1 mice spent as much time as Saline mice exploring the two objects (*q* = 0.97, *p* = 0.78 by Tukey’s test after one-way ANOVA; Fig 2C). However, the time MVK10 mice explored the objects was significantly shorter than that of Saline and MVK1 mice (Saline vs. MVK10, *q* = 5.8, *p* = 4.1×10^−3^; MVK1 vs. MVK10, *q* = 6.73, *p* = 1.7 ×10^−3^ by Tukey’s test after one-way ANOVA; Fig 2C). Based on these results, MVK10 mice were excluded from the test sessions since they did not fully explore the objects during training. Taken together with the decrease in bodyweight and locomotion in MVK10 mice, it is possible that 10 mg/kg of MVK administration ailed the mice, preventing them from exploring the objects.

During test sessions, Saline mice explored the relocated object more (*t* (4) = 5.7, *p* = 4.7×10^−3^ by one sample *t*-test; Fig 2F). However, the discrimination score of MVK1 mice was at chance level (*t* (4) = -0.24, *p* = 0.83 by one sample *t*-test; Fig 2F) and was lower than that of Saline mice (*t* (8) = 3.6, *p* = 6.6×10^−3^ by Student’s *t*-test; Fig 2F). MVK1 mice also showed an increase in the time exploring the objects compared to Saline mice (*t* (8) = -2.7, *p* = 0.034 by Student’s *t*-test; Fig 2E), which was caused by increased exploration of the object that remained at the familiar location (median_Saline_ = 5.9, median_MVK1_ = 11, *p* = 0.032 after Mann-Whitney Rank Sum Test). These data showed that MVK1 mice could not distinguish between the two objects, suggesting impaired spatial memory in MVK1 mice.

### MVK activates glial cells in the hippocampus

**Fig 3:**
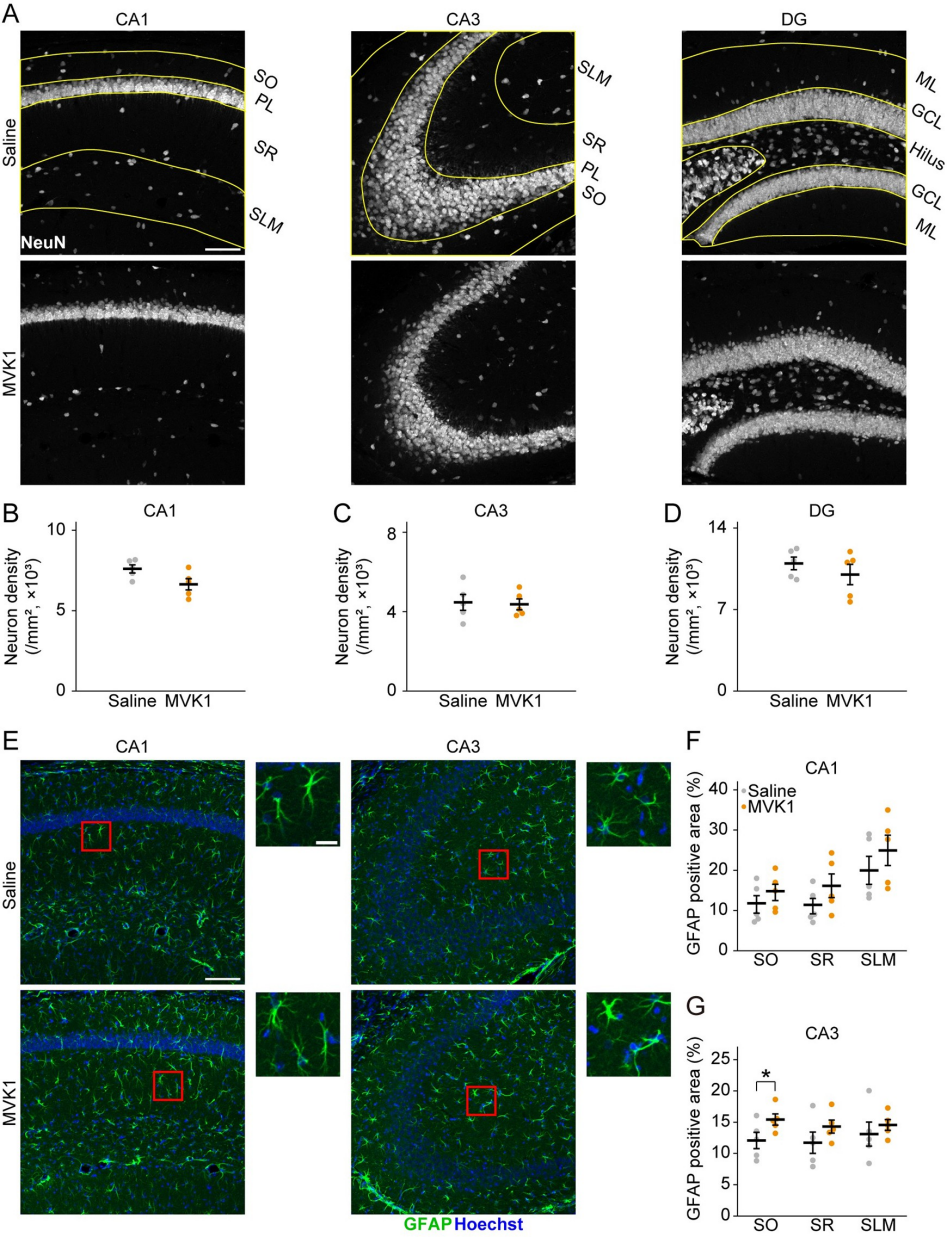
The influence of MVK on hippocampal neurons and astrocytes. (A) The representative confocal images of brain sections immunostained for NeuN (white). Yellow lines indicate the boundaries of each hippocampal layer. Scale bar = 100 μm. (B, C, and D) Neuron density in CA1 (B), CA3 (C), and DG (D) (N = 5 for each group). (E) The representative confocal images of brain sections immunostained for GFAP (green). The nuclei were labeled with Hoechst (blue). The areas within the red squares are magnified in the right columns. Scale bar = 100 μm. Scale bar in magnified image = 20 μm. (F and G) Percentage of GFAP-positive area in CA1 (C) and CA3 (D) (N = 5 for each group). *p < 0.05. The data are presented as mean ± SEM. Small dots indicate individual data points of each mouse. SO = stratum oriens; PL = pyramidal cell layer; SR = stratum radiatum; SLM = stratum lacunosum-moleculare; ML = molecular layer; GCL = granule cell layer.

Since the hippocampus is crucial for spatial memory, we focused on the changes of neurons and glial cells in the hippocampus induced by MVK administration via immunohistochemistry. The hippocampus has a layered structure: in the hippocampal CA1 and CA3 areas, the hippocampus is divided into the pyramidal cell layer, which is almost entirely composed of neurons, and stratum oriens (SO), stratum radiatum (SR) and stratum lacunosum-moleculare (SLM), which are enriched with glial cells (Fig 3A); hippocampal dentate gyrus (DG) consists of the granule cell layer, which is densely populated with excitatory neurons, the hilus and molecular layer (ML), which contains various types of cells including glial cells (Fig 3A). Since the components in each layer are different, we analyzed each layer separately. We evaluated the change to neurons by immunostaining NeuN, a marker for mature neurons. No neuronal loss was observed in the pyramidal cell layer of hippocampal CA1 or CA3 or the granule cell layer of the hippocampal DG (CA1, *t* (8) = 2.2, *p* = 0.057; CA3, *t* (8) = 0.2, *p* = 0.85; DG, *t* (8) = 0.93, *p* = 0.38 by Student’s *t*-test; Fig 3B–3D).

Next, we turned our attention to glial cells. Abundant evidence has shown that both microglia and astrocytes are closely involved in memory [14, 15]. We examined the changes in glial cells under the hypothesis that glial cells may be activated by MVK. First, we checked whether hippocampal astrocytes were activated in MVK1 mice. Reactive astrocytes are characterized by proliferation and increased expression of GFAP, an astrocyte marker for cell body and main processes [16]. Hence, in this study, we immunostained GFAP and measured the area covered by GFAP (Fig 3E). There was no significant increase in GFAP area in any layer of hippocampal CA1 after MVK administration (SO, *t* (8) = -1.0, *p* = 0.17; SR, *t* (8) = -1.4, *p* = 0.094; SLM, *t* (8) = -0.97, *p* = 0.18 by one-tailed Student’s *t*-test; Fig 3F). In CA3, the area of GFAP was not increased by MVK in SR and SLM but was significantly enlarged in SO (SO, *t* (8) = -2.1, *p* = 0.034; SR, *t* (8) = -1.3, *p* = 0.12; SLM, *t* (8) = -0.69, *p* = 0.26 by one-tailed Student’s *t*-test; Fig 3G). We did not measure the area of GFAP in the hippocampal DG since GFAP also labels neural progenitor cells in DG [17].

**Fig 4:**
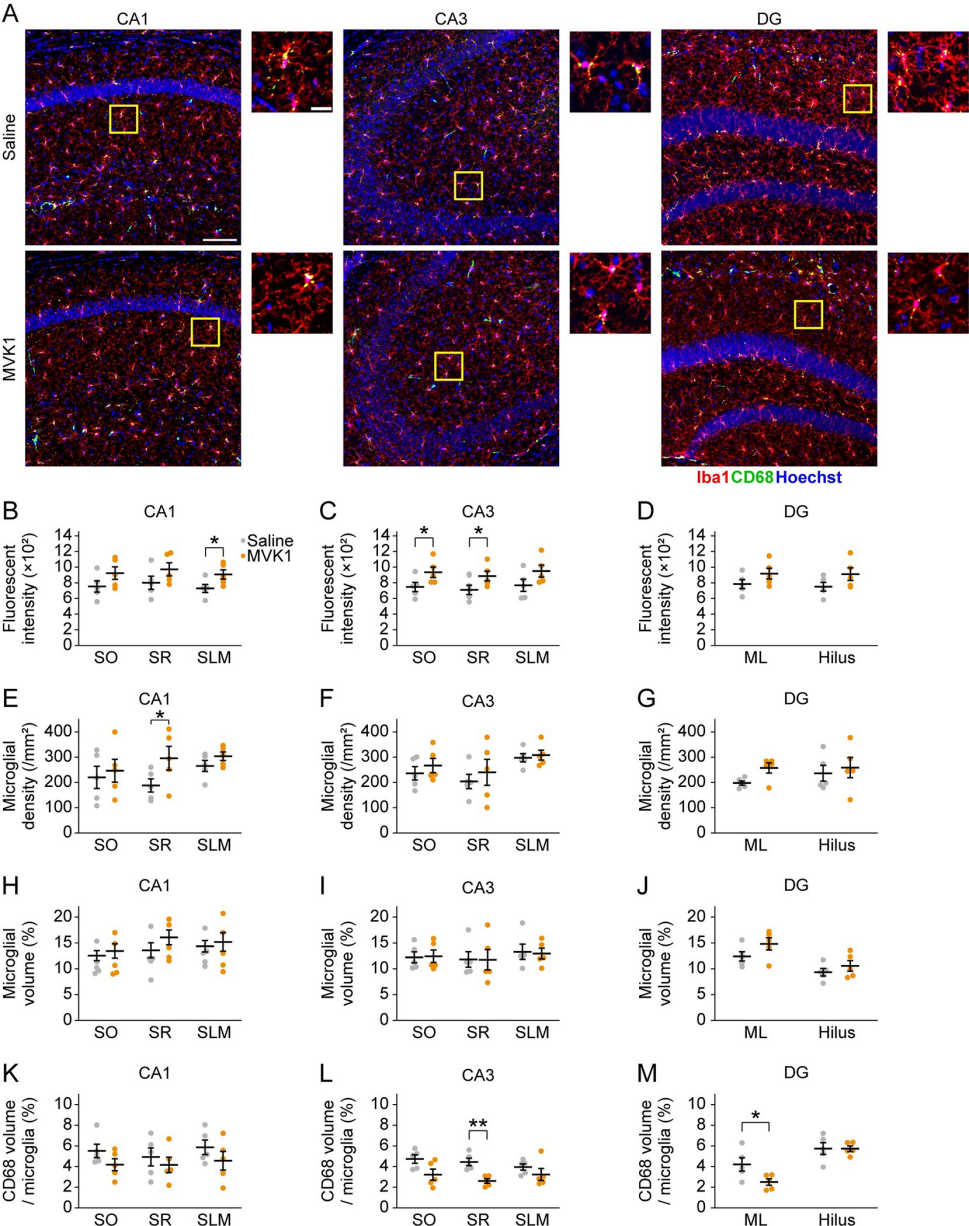
MVK activates microglia and decreases phagocytosis. (A) The representative confocal images of brain sections immunostained for Iba1 (red) and CD68 (green). The nuclei were labeled with Hoechst (blue). The areas within the yellow squares are magnified in the right columns. Scale bar = 100 μm. Scale bar in magnified image = 20 μm. (B, C, and D) Iba1 fluorescence intensity in CA1 (B), CA3 (C), and DG (D) (N = 5 for each group). (E, F, and G) Microglial density in CA1 (E), CA3 (F), and DG (G) (N = 5 for each group). (H, I, and J) Volume percentage of microglia in CA1 (H), CA3 (I), and DG (J) (N = 5 for each group). (K, L, and M) Volume percentage of CD68 in microglia in CA1 (K), CA3 (L), and DG (M) (N = 5 for each group). *p < 0.05. **p < 0.01. The data are presented as mean ± SEM. Small dots indicate individual data points of each mouse. SO = stratum oriens; SR = stratum radiatum; SLM = stratum lacunosum-moleculare; ML = molecular layer.

Then, we examined microglia by immunostaining Iba1, a cellular marker and an activation marker for microglia [18] (Fig 4A). No significant increase in Iba1 fluorescence intensity was observed in SO and SR of hippocampal CA1, but the fluorescence intensity was significantly increased in SLM of MVK1 mice compared to Saline mice (SO, *t* (8) = -1.6, *p* = 0.077; SR, *t* (8) = -1.5 *p* = 0.093; SLM, *t* (8) = -2.3, *p* = 0.027 by one-tailed Student’s *t*-test; Fig 4B). In CA3, Iba1 fluorescence intensity was not significantly increased in SLM but was significantly increased by MVK in SO and SR (SO, *t* (8) = -2.1, *p* = 0.035; SR, *t* (8) = -2.0, *p* = 0.039; SLM, *t* (8) = -1.7, *p* = 0.066 by one-tailed unpaired *t*-test; Fig 4C). In the hippocampal DG, there was no significant increase in Iba1 fluorescence intensity in MVK1 mice compared to Saline mice (Fig 4D).

Microglia activation is also characterized by its proliferation [18]. Therefore, we calculated the density of microglia in each layer of the hippocampus. We found no significant increase in microglial density in CA1 SO and SLM, but the microglia density was significantly higher in the CA1 SR of MVK1 mice (SO, *t* (8) = -0.42, *p* = 0.34; SR, *t* (8) = -2.0, *p* = 0.040; SLM, *t* (8) = -1.4, *p* = 0.10 by one-tailed unpaired *t*-test; Fig 4E). There was no MVK-induced increase in microglia density in hippocampal CA3 (SO, *t* (8) = -0.80, *p* = 0.22; SR, *t* (8) = -0.62, *p* = 0.28; SLM, *t* (8) = -0.42, *p* = 0.35 by one-tailed unpaired *t*-test; Fig 4F) or DG (ML, median_Saline_ = 2.0×10^2^, median_MVK1_ = 2.7×10^2^, *p* = 0.095 after Mann-Whitney Rank Sum Test; Hilus, *t* (8) = -0.44, *p* = 0.34 by one-tailed unpaired *t*-test; Fig 4G).

Finally, the volume of microglia was also measured. However, the volume percentage of microglia did not increase in all the hippocampal layers (CA1 SO, *t* (8) = -0.53, *p* = 0.31; CA1 SR, *t* (8) = -1.2, *p* = 0.13; CA1 SLM, *t* (8) = -0.39, *p* = 0.35 by one-tailed unpaired *t*-test; Fig 4H; CA3 SO, median_Saline_ = 0.11, median_MVK1_ = 0.11, *p* = 1.0 after Mann-Whitney Rank Sum Test; CA3 SR, *t* (8) = 0.026, *p* = 0.49; CA3 SLM, *t* (8) = 0.19, *p* = 0.43 by one-tailed unpaired *t*-test; Fig 4I; ML, *t* (8) = -1.6, *p* = 0.071; Hilus, *t* (8) = -0.97, *p* = 0.18 by one-tailed unpaired *t*-test; Fig 4J).

In summary, we have found an increase of GFAP area in CA3 SO, increases of Iba1 intensity in CA1 SLM, CA3 SO and CA3 SR, and increased microglia density in CA1 SR. GFAP area, Iba1 intensity and microglia density observed in other hippocampal regions also exhibited tendencies to increase. These results suggest that astrocytes and microglia in several hippocampal subregions were activated by MVK administration.

Furthermore, we measured the volume percentage of CD68, a microglial lysosome-associated protein, to evaluate the phagocytic function of microglia [18]. No significant change in the volume percentage of CD68 was found in SO, SR, and SLM of hippocampal CA1 (SO, *t* (8) = 1.5, *p* = 0.17; SR, *t* (8) = 0.67, *p* = 0.52; SLM, *t* (8) = 1.1, *p* = 0.29 by two-tailed unpaired *t*-test; Fig 4K). In CA3, although there were no significant CD68 volume changes in SO and SLM, CD68 volume percentage was significantly reduced in MVK1 mice compared to Saline mice in SR (SO, *t* (8) = 2.3, *p* = 0.055; SR, *t* (8) = 4.4, *p* = 2.4×10^−3^; SLM, *t* (8) = 1.1, *p* = 0.30 by two-tailed unpaired *t*-test; Fig 4L). In hippocampal DG, CD68 volume was significantly reduced in ML in MVK1 mice (ML, *t* (8) = 2.4, *p* = 0.045; Hilus, *t* (8) = 0.018, *p* = 0.99 by two-tailed unpaired *t*-test; Fig 4M). The analysis on the microglial CD68 indicates that MVK impaired the phagocytic function of microglia. Therefore, our findings show that systemic MVK administration impaired spatial memory and also altered glial functions in hippocampal subregions.

## Discussion

In the current study, we have shown that systemic exposure to MVK, a hazardous environmental pollutant, for one week can cause memory deficits accompanied by activated glial cells and altered glial functions in hippocampal subregions. Astrocyte was activated by MVK in CA3 SO. MVK administration activated microglia in CA1 SR, CA1 SLM, CA3 SO, and CA3 SR. Otherwise, the activity of CD68 decreased by MVK in CA3 SR and DG ML. Higher dosages of MVK (10 mg/kg and 100 mg/kg) were either deadly or harmful to the health of administered mice (Fig 1). On the other hand, 1 mg/kg MVK administration impaired hippocampus-dependent memory without affecting weight gain and locomotion (Figs 1 and 2). We also observed increased GFAP area, Iba1 intensity, microglia density, and decreased volume percentage of CD68 in the hippocampus of MVK1 mice, which indicates the activation and functional changes of glial cells (Figs 3 and 4).

As a highly unstable oxidant, MVK has been shown to impair the survival of cultured cells by depleting antioxidants. Such oxidative stress is known to activate the proinflammatory pathways of both astrocytes and microglia, which in turn may produce reactive oxygen species (ROS) to exacerbate the influence of MVK [19]. Notably, proinflammatory microglia are considered the major source of ROS in the neurodegenerative brain and the key activator of reactive astrocytes [20, 21].

Accumulating evidence points to the fact that activated glial cells can impair memory. Reactive astrocytes have been shown to interfere with learning and memory through GABA and TNFα signals [16, 22]. Similarly, activated microglia are known to eliminate synapses through the complement pathway, which causes memory deficits [23, 24]. However, in this study, although we have observed indications for microglia activation including increased Iba1 intensity and microglia density, phagocytic activity appeared to be downregulated in MVK1 mice since the volume percentage of CD68 was decreased. These data resemble the results of previous studies on microglial changes in chronic alcohol administration and a model of multiple sclerosis [25, 26]. Microglia activation and decreased CD68 expression were observed in these circumstances, both of which exhibit memory deficits as well [27, 28]. Therefore, it would be interesting to elucidate whether downregulated microglial phagocytic activity can impair memory.

In addition, we did not observe any neuronal loss in our experiments. However, it is possible that MVK impairs memory by disrupting neuronal functions. Previous study has shown that prolonged exposure to another hazardous oxidant, acrolein, can decrease synaptic glutamate release [29].

Overall, our findings uncovered the influence of MVK exposure on memory and revealed that MVK administration could activate glial cells and alter glial functions, which is a probable cause for memory impairment. Since MVK exists in our surroundings, further studies are required to examine the effect of MVK on healthy and diseased brains at functional and cellular levels.

## Supporting information

S1 File (XLSX)
